# Application value of combination therapy of periodontal curettage and root planing on moderate-to-severe chronic periodontitis in patients with type 2 diabetes

**DOI:** 10.1186/s13005-020-00253-z

**Published:** 2021-04-08

**Authors:** Yonghuan Bian, Changhao Liu, Zhaojiang Fu

**Affiliations:** 1 Department of Stomatology, Linyi Central Hospital, Linyi, Shandong China; 2 Department of Stomatology, Weifang People’s Hospital, Weifang, Shandong China; 3 Department of Periodontology, Qingdao Stomatological Hospital, No. 17 Dexian Road, Qingdao, 266000 Shandong China

**Keywords:** Chronic periodontitis, Periodontal curettage, Root planing, Inflammatory factors, Type 2 diabetes

## Abstract

**Background:**

Our study attempted to observe the value of periodontal curettage combined with root planing on moderate-to-severe chronic periodontitis in patients with type 2 diabetes.

**Methods:**

There involved 72 patients with type 2 diabetes mellitus complicated with moderate-to-severe chronic periodontitis who were diagnosed and treated in our hospital from January 2019 to December 2019. The patients enrolled were randomly divided into four groups using a computer-generated table: root planing and periodontal curettage combined group (*n* = 18), root planning group (*n* = 18), periodontal curettage group (*n* = 18) and cleansing group (*n* = 18). Blood glucose, plaque index (PI), gingival index (GI), probing depth (PD), attachment loss (AL), serum levels of inflammatory factors (Tumor Necrosis Factor Alpha [TNF- α] and hypersensitive C-reactive protein [hs-CRP]) were observed before and after treatment. The collecting dates were analyzed by the chi-square χ 2 test, repeated measurement analysis of variance, or t-test according to different data types and research objectives.

**Results:**

Before treatment, there was no significant difference in PI, GI, PD and AL among the four groups (*P*> 0.05), while after 3-month treatment, the levels of PI, GI, PD and AL in the combined group were lower than those in the root planing group, periodontal curettage group and cleansing group, with both root planing group and periodontal curettage group significantly lower than cleansing group (*P*< 0.05). The fasting blood glucose, 2-h postprandial blood glucose and glycosylated hemoglobin in the combined group, root planing group, periodontal curettage group and cleansing group were significantly lower than those before treatment (*P* < 0.05). Before treatment, there was no significant difference in TNF- α and hs-CRP among the four groups (*P*> 0.05), but the levels of TNF- α and hs-CRP in the four groups decreased significantly after 3-month treatment (*P*< 0.05). The levels of TNF- α and hs-CRP in the combined group were lower than those in the root planing group, periodontal curettage group and cleansing group, and those in the root planing group and periodontal curettage group were significantly lower than those in the cleansing group (*P*< 0.05).

**Conclusion:**

The combination therapy of periodontal curettage and root planing exerted beneficial effects on moderate-to-severe chronic periodontitis in patients with type 2 diabetes mellitus, which holds the potential to maintain the level of blood glucose and improve the quality of life of the patients.

## Background

Chronic periodontitis is a common clinical disease. It is acknowledged as a chronic inflammation of periodontal supporting tissue caused by local factors, which usually occurs in patients over 35 years old [1, 2]. Diabetes is a common chronic systemic metabolic disease characterized by hyperglycemia. According to related research reports [3], the global prevalence rate of diabetes has experienced an increased from about 108 million in 1980 to 451 million in 2017. And around 693 million people are expected to be diagnosed with diabetes in 2045, of which type 2 diabetes accounts for more than 90%. Chronic inflammation is believed to be in the pathogenesis of type 2 diabetes (DM) [4, 5].

Periodontitis is a cause of systemic inflammation [6, 7]. Compared with individuals without diabetes, individuals with diabetes have a higher risk of developing chronic periodontitis and more severe chronic periodontitis [8–11]. As high as 72.79% of patients with type 2 diabetes are prone to chronic periodontitis. Patients with type 2 diabetes mellitus complicated with chronic periodontitis could suffer from respiratory, cardio-cerebrovascular and other serious complications, and even death in serious cases. Therefore, the therapy approach for patients with type 2 diabetes mellitus complicated with chronic periodontitis is one of the key topics in clinical research in recent years. A variety of protocols are used for prevention and treatment of periodontitis including basic treatment (e.g. periodontal curettage and root planning) and guided tissue regeneration [12]. Periodontal curettage can control the progression of periodontitis and prevent the further loss of periodontal support tissue [13]. Root planing is widely used procedures for treatment of periodontitis, due to their low cost and effectiveness in reducing the clinical signs of inflammation and the levels of pathogens [14, 15].

In this study, 62 patients with type 2 diabetes mellitus complicated with moderate-to-severe chronic periodontitis were enrolled to observe the effect of periodontal curettage combined with root planing in patients with type 2 diabetes mellitus complicated with moderate-to-severe chronic periodontitis, to offer insight for clinical practice.

## Methods

### Clinical background

There involved a total of 72 patients with type 2 diabetes mellitus complicated with moderate-to-severe chronic periodontitis who were diagnosed and treated in the Department of Endocrinology and Stomatology of our hospital from January 2019 to December 2019. The patients were randomly divided into four groups using a computer-generated table: root planing and periodontal curettage combined group (*n* = 18), root planing group (*n* = 18), periodontal curettage group (*n* = 18) and cleansing group (*n* = 18). The patients in the combined group included 7 males and 11 females, 35–72 years old, with an average age of (61.83 ±7.21). There were 6 males and 12 females in the root planing group, 36–71 years old, with an average age of (61.37 ±7.45). The periodontal curettage group included 5 males and 13 females, 37–73 years old, with an average age of (61.95 ±7.36). There were 7 males and 12 females in the cleansing group, 38–72 years old, with an average age of (61.69 ±7.31). There was no significant difference in general data among the above four groups (*P* > 0.05). The formulation of this research program is in line with the relevant requirements of the Helsinki Declaration of the World Medical Association.

### Diagnostic criteria

#### Diagnostic criteria for type 2 diabetes mellitus

All patients met the diagnostic criteria of type 2 diabetes established by WHO in 1999, with 2-h postprandial blood glucose ≥ 11.1 mmol/L and fasting blood glucose ≥ 7.0 mmol/L.

### Diagnostic criteria of periodontitis

In the context of the 2017 World Workshop, it is suggested that a patient is a periodontitis case in the context of clinical care if: 1.Interdental clinical attachment loss (CAL) is detectable at ≥2 non-adjacent teeth, or 2.Buccal or oral CAL ≥3 mm with pocketing > 3 mm is detectable at ≥2 teeth; and the observed CAL cannot be ascribed to non-periodontal causes [4]. On this basis, a patient was diagnosed as moderate-to-severe periodontitis when met the following conditions: More than 30% of the affected teeth surface had probing depth ≥ 4 mm or one molar had root bifurcation lesions of more than degree I accompanied with probing ≥ 3 mm or at least one affected tooth with probe depth ≥ 5 min.

### Selection criteria

Inclusion criteria: patients met the above diagnostic criteria; over 35 years old; complete clinical data; no coagulation disorder; no antibiotics within 3 months; no periodontal treatment within 1 year; no history of smoke; all patients and their families voluntarily participated in the study and signed informed consent. Exclusion criteria: less than 2 years of estimated survival; periodontitis patients who needed surgery or root canal therapy; acute complications of diabetes; pregnancy or lactation; systemic diseases with periodontal therapy.

### Treatment methods

All patients were given oral hygiene guidance and oral health education by the attending physician of stomatology department of our hospital, including how to correctly identify pigments, dental plaque, dental calculus and soft dirt, etc.

According to each patient’s gender, age, body mass, labor intensity and course of disease, psychological and dietary habits, personalized diabetes diets were formulated for each patient to guide patients to eat scientifically and healthily. We also taught patients to have healthy exercise: walking for about 30 min 1 h after meals, and exercising at least 5 times a week. Then we help patients develop scientific exercise plans. Patients were informed to use Bass brushing method, and dental tools should be selected according to the oral characteristics of patients with brushing time ≥ 3 min, etc., according to the diagnosis and treatment of chronic periodontitis regular research manual. In the combined group, root planing combined with periodontal curettage therapy was given on the basis of the abovementioned health education. In the root planing group, root planing treatment was given on the basis of the above health education. In the periodontal curettage group, periodontal curettage treatment was given on the basis of the above health education. In the cleansing group, cleansing treatment was given as follows: cleaning up scales and plaque bacteria on patients’ gingiva, subgingival cleaning of patients’ periodontal pockets and polishing. All the above treatments were completed by one deputy chief physician and one attending doctor in our hospital. All patients maintained the original treatment plan for diabetes during the observation period.

### Observation indexes

Blood glucose index: the changes of fasting blood glucose, 2-h postprandial blood glucose and glycosylated hemoglobin in all patients before and after treatment were recorded.

Periodontal clinical index: 1) the plaque index (PI) was scored according to the amount of plaque, with the score of 0–3. 0 point: no plaque in the marginal gingival area; 1 point: no plaque in the visual examination, but a small amount of plaque can be scraped in the adjacent gingiva or free gingiva with the probe; 2 points: there are a small amount of soft deposits in the gingival pocket, free gingival area or adjacent tooth surface; 3 points: there are a lot of soft deposits in the gingival pocket or free gingival area and adjacent dental surface. 2) The gingival index (GI) was scored according to the gingival condition, with a score of 0–3. 0 point: healthy gums; 1 point: mild gingival inflammation, no bleeding gums by probing, slight changes in gingival color and mild edema; 2 points: moderate gingival inflammation, bleeding or red gums with light edema by probing; 3 points: severe gingival inflammation, gum with automatic bleeding tendency, obvious redness, swelling or ulcer. 3) The probing depth (PD) was observed by WHO probe for periodontal pocket, and the whole number was taken. 4) Attachment loss (AL). 5) The levels of serum inflammatory factors (Tumor Necrosis Factor Alpha (TNF- α) and hypersensitive C-reactive protein (hs-CRP)) were calculated before and 3 months after treatment. 6) The questionnaire made by modifying the existing questionnaire (diabetes-specific quality of life questionnaire-Chinese version [16]) was used to assess the patients’ quality of life. The questionnaire included 4 dimensions (therapeutic dimension, social relationship, psychological (mental) and physiological function) and 24 items. It rates responses on a 5-point-Likert scale [17, 18] (0=always, 1=frequently, 3=sometimes, 4= infrequently, 5=never). The constituent items were summed to calculate the total score (120 points). The high score represents a higher quality of life of the patients.

### Statistical analysis

All the data in this study were analyzed by IBM Microsoft SPSS 21.0software. The counting data were expressed by rate (%) and compared by chi-square χ 2 test; $\overline{x}$ ±s was used to represent the measurement data; repeated measurement variance was used to analyze the overall comparison of each group data, and t-test was used for the pairwise comparison of inter-group and intra-group data. *P*< 0.05 represented significant difference.

## Results

### General information

There was no significant difference in the general data among the combined group, the root planing group, the periodontal curettage group and the cleansing group (*P* > 0.05), as shown in Table 1.

**Table 1:** General information

Item	Combined group (*n*=18)	Root planing group (*n*=18)	Periodontal curettage group (*n*=18)	Cleansing group (*n*=18)	*χ*^*2*^*/t*	*P*
Sex (n)						
Female/male	7/11	6/12	5/12	7/12	0.12	0.729
Age (year, $\overline{x}$ ±s)	61.83±7.21	61.37±7.45	61.95±7.36	61.69±7.31	0.021	0.996
BMI (kg/m^2^, $\overline{x}$ ±s)	24.45±2.13	24.32±2.04	24.29±1.98	24.39±2.28	0.032	0.874
Course of diabetes (year, $\overline{x}$ ±s)	5.09±1.09	5.07±1.11	5.12±1.23	5.17±1.09	0.027	0.094

### Comparison of periodontal clinical indexes among the four groups

Before treatment, there was no significant difference in PI, GI, PD and AL among the four groups (*P*> 0.05), while after 3-month treatment, the levels of PI, GI, PD and AL in the combined group were lower than those in the root planing group, periodontal curettage group and cleansing group, and those in the root planing group and periodontal curettage group were significantly lower than the cleansing group (*P*< 0.05)(Table. 2).

**Table 2:** Comparison of periodontal clinical indexes among the four groups ($\overline{x}$ ±s)

Group	Time	PI (mm)	GI (mm)	PD (mm)	AL (mm)
Combined group (*n*=18)	Before therapy	2.49±0.63	0.96±0.12	5.66±0.59	5.78±1.72
	After 3 months of therapy	0.41±0.06	0.32±0.08	3.01±0.19	3.28±0.28
Root planing group (*n*=18)	Before therapy	2.38±0.71	0.99±0.16	5.62±0.37	5.68±1.01
	After 3 months of therapy	0.54±0.02	0.46±0.09	3.42±0.15	3.49±0.32
Periodontal curettage group (*n*=18)	Before therapy	2.45±0.59	0.92±0.14	5.61±0.37	5.82±1.39
	After 3 months of therapy	0.52±0.07	0.47±0.10	3.40±0.16	3.51±0.33
Cleansing group (*n*=18)	Before therapy	2.51±0.63	0.98±0.17	5.71±0.37	5.87±1.28
	After 3 months of therapy	0.64±0.06	0.54±0.07	3.76±0.34	3.65±0.53
*t*		0.144	0.78	0.177	0.062
*P*		0.933	0.509	0.912	0.98
*t’*		176.034	20.796	33.883	2.939
*P′*		<0.001	<0.001	<0.001	0.039

Note: t and P were the comparison of four groups before treatment, and t’ and P′ were the comparison of four groups after 3 months of treatment

### Comparison of blood glucose levels among the four groups

Before treatment, there was no significant difference in fasting blood glucose, 2-h postprandial blood glucose and glycosylated hemoglobin among the four groups (*P* > 0.05). After 3 months of treatment, the fasting blood glucose, 2-h postprandial blood glucose and glycosylated hemoglobin in the combined group, root planing group, periodontal curettage group and cleansing group were significantly lower than those before treatment (*P* < 0.05). However, no significant difference was found in fasting blood glucose, 2-h postprandial blood glucose and glycosylated hemoglobin among the four groups (*P* > 0.05) (Table. 3).

**Table 3:** Comparison of blood glucose levels among four groups ($\overline{x}$ ±s)

Group	Time	Fasting blood glucose (mmol/L)	2-h postprandial blood glucose (mmol/L)	Glycosylated hemoglobin (%)
Combined group (*n*=18)	Before therapy	9.98±1.28	9.29±1.01	9.83±1.78
	After 3 months of therapy	8.03±1.03	8.13±1.07	7.38±0.87
Root planing group (*n*=18)	Before therapy	9.94±1.32	9.31±1.17	9.81±1.82
	After 3 months of therapy	8.23±1.23	8.23±1.73	7.42±0.65
Periodontal curettage group (*n*=18)	Before therapy	9.97±1.42	9.30±1.35	9.79±1.29
	After 3 months of therapy	8.16±1.22	8.32±0.76	7.68±1.73
Cleansing group (*n*=18)	Before therapy	9.92±1.39	9.34±1.46	9.85±1.82
	After 3 months of therapy	8.21±1.43	8.65±0.87	7.76±1.27
*t*		0.007	0.005	0.004
*P*		0.999	1.0	1.0
*t’*		0.095	0.669	0.441
*P′*		0.963	0.574	0.724
t^*^		5.036	3.345	4.283
P^*^		<0.001	0.002	<0.001
*t*^*#*^		3.886	2.194	5.247
*P*^*#*^		<0.001	0.035	<0.001
t^*#^		4.12	2.684	4.148
P^*#^		<0.001	0.011	<0.001
t^*#*^		3.638	2.087	3.995
P^*#*^		0.001	0.044	<0.001

Note: t and P were the comparison of four groups before treatment; t’ and P′ were the comparison of four groups 3 months after treatment; t* and P* were the comparison of combined group before and 3 months after treatment; t# and P# were the comparison of root planing group before and 3 months after treatment; t*# and P*# were the comparison of periodontal curettage group before and 3 months after treatment; t*#* and P*#* were the comparison of cleansing group before and 3 months after treatment

### Comparison of TNF- α and hs-CRP among the four groups

Before treatment, there was no significant difference in TNF- α and hs-CRP among the four groups (*P*> 0.05), while the levels of TNF- α and hs-CRP in the four groups decreased significantly after 3 months of treatment (*P*< 0.05). The levels of TNF- α and hs-CRP in the combined group were lower than those in the root planing group, periodontal curettage group and cleansing group, and the levels of TNF- α and hs-CRP in the root planing group and periodontal curettage group were significantly lower than those in the cleansing group (*P*< 0.05), as shown in Table 4.

**Table 4:** Comparison of TNF- α and hs-CRP among four groups ($\overline{x}$ ±s)

Group	Time	TNF-α (pg/ml)	hs-CRP (mg/L)
Combined group (*n*=18)	Before therapy	6.09±1.34	5.87±1.63
	After 3 months of therapy	1.98±0.32	1.87±0.28
Root planing group (*n*=18)	Before therapy	5.98±1.23	5.81±1.69
	After 3 months of therapy	2.63±0.21	2.86±0.65
Periodontal curettage group (*n*=18)	Before therapy	5.99±1.42	5.79±1.76
	After 3 months of therapy	2.73±0.42	2.71±0.59
Cleansing group (*n*=18)	Before therapy	5.96±1.56	5.89±1.81
	After 3 months of therapy	2.93±0.17	2.84±0.65
*t*		0.031	0.014
*P*		0.993	0.998
*t’*		34.579	12.582
*P′*		<0.001	<0.001
t^*^		12.657	10.261
t^*^		<0.001	<0.001
P^*^		11.39	6.912
*t*^*#*^		<0.001	<0.001
*P*^*#*^		9.34	7.04
t^*#^		<0.001	<0.001
P^*#^		8.192	6.728
t^*#*^		<0.001	<0.001

Note: t and P were the comparison of four groups before treatment; t’ and P′ were the comparison of four groups 3 months after treatment; t* and P* were the comparison of combined group before and 3 months after treatment; t# and P# were the comparison of root planing group before and 3 months after treatment; t*# and P*# were the comparison of periodontal curettage group before and 3 months after treatment; t*#* and P*#* were the comparison of cleansing group before and 3 months after treatment

### Comparison of diabetes-specific quality of life scores among the four groups

Before treatment, there was no significant difference in treatment dimension, social relations, psychological (mental) and physiological function among the four groups (*P* > 0.05), but after 3 months of treatment, the treatment dimensions, social relations, psychological (mental) and physiological functions of the four groups were significantly lower than those before treatment (*P* < 0.05). The scores of treatment dimension, social relations, psychological (mental) and physiological function in the combined group were significantly higher than those in the root planing group, periodontal curettage group and cleansing group (*P* < 0.05). However, no significant difference was observed in the scores of treatment dimension, social relations, psychological (mental) and physiological function when comparing root planing group, periodontal curettage group and cleansing group (*P* > 0.05), as shown in Table 5.

**Table 5:** Comparison of diabetes-specific quality of life scores among the four groups ($\overline{x}$ ±s)

Group	Time	Treatment dimension	Social relations	Psychological (mental) condition	Physiological function
Combined group (*n*=18)	Before therapy	14.09±1.29	20.18±2.38	18.93±3.28	17.63±2.98
	After 3 months of therapy	35.98±3.82	25.47±3.31	24.39±0.87	26.47±0.82
Root planing group (*n*=18)	Before therapy	14.28±1.31	20.27±2.29	19.03±3.52	17.67±2.77
	After 3 months of therapy	30.01±3.18	22.19±3.17	22.01±2.71	24.39±2.18
Periodontal curettage group (*n*=18)	Before therapy	14.16±2.01	20.12±2.19	19.01±3.76	17.70±2.38
	After 3 months of therapy	29.97±3.09	22.21±3.08	22.05±3.48	24.41±3.27
Cleansing group (*n*=18)	Before therapy	14.21±1.82	20.25±2.43	19.11±2.14	17.75±2.84
	After 3 months of therapy	29.73±2.17	22.87±3.07	22.12±2.87	24.08±3.12
*t*		0.043	0.016	0.009	0.006
*P*		0.988	0.997	0.999	0.999
*t’*		17.088	4.366	3.44	3.362
*P′*		<0.001	0.007	0.022	0.024
t^*^		−23.034	−5.505	−6.826	−12.135
P^*^		<0.001	<0.001	<0.001	<0.001
*t*^*#*^		−19.404	−2.083	−2.369	−8.088
*P*^*#*^		<0.001	<0.001	0.023	<0.001
t^*#^		−18.196	−2.346	− 2.517	−7.039
P^*#^		<0.001	0.025	0.017	<0.001
t^*#*^		−23.249	− 2.839	−3.567	−6.365
P^*#*^		<0.001	0.007	0.001	<0.001

Note: t and P were the comparison of four groups before treatment; t’ and P′ were the comparison of four groups 3 months after treatment; t* and P* were the comparison of combined group before and 3 months after treatment; t# and P# were the comparison of root planing group before and 3 months after treatment; t*# and P*# were the comparison of periodontal curettage group before and 3 months after treatment; t*#* and P*#* were the comparison of cleansing group before and 3 months after treatment

## Discussion

Chronic periodontitis is a chronic inflammation of periodontal tissue, characterized by periodontal tissue inflammation, attachment loss and alveolar bone destruction [19]. The disease process of chronic periodontitis is affected by respiratory diseases, cardiovascular diseases, diabetes and many other systemic diseases, among which chronic periodontitis is closely related to type 2 diabetes and insulin resistance [20]. Chronic periodontitis has been currently regarded as one of the complications of diabetes, which has impact on the control of blood glucose [20]. Additionally, patients with type 2 diabetes complicated with chronic periodontitis suffer from long-term hyperglycemia that could lead to bacterial growth and infection in the oral cavity. Hence there is a high incidence of chronic periodontitis in patients with type 2 diabetes [21, 22]. In addition, type 2 diabetic patients with chronic periodontitis can cause inflammatory reaction and immune response, and promote the disorder of glucose metabolism in the body [20, 23]. Thus, it is crucial to adopt active and effective treatment measures to control the blood glucose level and inflammatory reaction in patients with type 2 diabetes mellitus complicated with chronic periodontitis.

At present, periodontal curettage, root planing and cleansing are commonly used in the treatment of moderate-to-severe chronic periodontitis, which can remove pathogenic factors, such as food impaction, soft dirt, dental calculus, plaque attached to periodontal bags, and so on [24]. The approaches could treat local inflammation by destroying the living environment of periodontal pathogenic bacteria. As a matter of fact, no specific guidelines exist for the treatment of chronic periodontitis in patients with type 2 diabetes. And there is little clinical application of periodontal curettage combined with root planing in patients with type 2 diabetes complicated with moderate-to-severe chronic periodontitis. For the reasons mentioned above, we attempt to explore the application value of combination therapy of periodontal curettage and root planing on moderate-to-severe chronic periodontitis in patients with type 2 diabetes.

Van Steenberghe et al. [25] suggested that minocycline hydrochloride ointment combined with one-stop subgingival curettage and root planing can effectively control plaque in patients with moderate chronic periodontitis. According to Fu et al. [26], valaciclovir combined with subgingival curettage and root planing was effective in the treatment of severe chronic periodontitis. Sindhura et al. [27] used two different subgingival curettage combined with root planing and effectively improved the plaque index and periodontal probing depth in patients with type 2 diabetes and chronic periodontitis. The results of our study showed that after 3 months of treatment, the PI, GI, PD and AL in the combined group were lower than those in the root planing group, periodontal curettage group and cleansing group, and those in the root planing group and periodontal curettage group were lower than those in the cleansing group. It was suggested that periodontal curettage combined with root planing therapy can effectively improve the gingival index and reduce the plaque index, probing depth and attachment loss in patients with type 2 diabetes mellitus complicated with moderate-to-severe chronic periodontitis [28]. The results also demonstrated that after 3 months of treatment, the fasting blood glucose, 2-h postprandial blood glucose and glycosylated hemoglobin in the combined group, root planing group, periodontal curettage group and cleansing group were lower than those before treatment, but without significant difference among the four groups. It was suggested that periodontal curettage combined with root planing, periodontal curettage, root planing and cleansing therapy were not associated with the blood glucose level of patients with type 2 diabetes complicated with moderate-to-severe chronic periodontitis, which is similar to the results of previous studies [29].

TNF- α is produced by monocytes and macrophages with a variety of biological activities, which can promote T cells to produce a variety of inflammatory factors, and then promote the occurrence of inflammatory response [30]. CRP is an acute phase reactive protein synthesized by the liver stress state, which contains five polypeptide chain subunits. TNF- α and CRP are commonly used inflammatory markers in clinic and are positively correlated with the pathogenesis of diabetes [31]. Martínez-Aguilar et al. [32] confirmed that the level of glycosylated hemoglobin was positively correlated with the level of TNF- α in patients with type 2 diabetes complicated with moderate-to-severe chronic periodontitis. According *the Expert Consensus on the goal of HbA1c Control of Type 2 Diabetes in Chinese Adults* formulated by the Endocrinology Branch of Chinese Medical Association in 2011, type 2 diabetes patients with HbA1c ≥ 7.0% represent the ineffective control of blood glucose, which could increase the incidence of cardiovascular events and aggravate the condition of patients with chronic periodontitis [33]. CRP is regarded as one of the risk factors for chronic periodontitis. And periodontal infection will lead to an increase in systemic inflammation, hyperglycemia, insulin resistance and advanced-glycation end products, and further aggravate tissue degradation, degradation and proliferation. Therefore, the reduced levels of TNF- α and CRP in patients with type 2 diabetes mellitus complicated with moderate-to-severe chronic periodontitis are linked to the balance of blood glucose. Baser et al. [34] indicated that there was a significant positive correlation between hs-CRP and the severity of periodontitis in patients with type 2 diabetes mellitus with chronic periodontitis. Based on our results, after 3 months of treatment, the levels of TNF- α and hs-CRP in the four groups were lower than those before treatment. The levels of TNF- α and hs-CRP in the combined group were lower than those in the root planing group, periodontal curettage group and cleansing group, and the levels of TNF- α and hs-CRP in the root planing group and periodontal curettage group were lower than those in the cleansing group. It was suggested that periodontal curettage combined with root planing therapy can effectively reduce the levels of TNF- α and hs-CRP in patients with type 2 diabetes mellitus complicated with moderate-to-severe chronic periodontitis [35]. Hypothetically, the effective removal of microorganisms in dental plaque and periodontal pocket can regulate blood glucose, thus reducing the level of serum inflammation.

Diabetes-specific quality of life questionnaire is a special questionnaire, which has been widely used to evaluate the quality of life of patients with diabetes. According to study supported by Huang et al. [36], diabetes-specific quality of life questionnaire can effectively evaluate the quality of life of elderly patients with type 2 diabetes. Thomsen et al. [37] studied the effect of mindfulness decompression therapy on the quality of life of patients with type 2 diabetes mellitus complicated with anxiety in community. The current study suggested that after 3 months of treatment, the scores of treatment dimension, social relations, psychological (mental) and physiological function in the combined group were higher than those in the root planing group, periodontal curettage group and scaling group, indicating that periodontal curettage combined with root planing therapy can effectively improve the quality of life of patients with type 2 diabetes mellitus complicated with moderate-to-severe chronic periodontitis. Through analysis of our study, considering that cleansing therapy is a relatively simple periodontal therapy and can also be carried out in the community, it is suggested to promote cleansing therapy as an effective intervention among patients with type 2 diabetes in the community. Given the small sample size included in this study with 3-month follow-up, further studies with larger sample size and longer follow-ups are warranted.

## Conclusion

Taken together, the combination therapy of periodontal curettage and root planing therapy elicited valid effects in terms of reducing PI, GI, PD and AL, stabilizing blood glucose level, reducing serum inflammatory factors, and improving quality of life in patients with type 2 diabetes mellitus complicated with moderate-to-severe chronic periodontitis. Hence the combination is available for wide clinical application.

## Data Availability

The datasets used and/or analysed during the current study are available from the corresponding author on reasonable request.

## Abbreviations

AL: attachment loss
GI: gingival index
PD: probing depth
PI: plaque index
